# A Missense Mutation in the Collagen Triple Helix of *EDA* Is Associated with X-Linked Recessive Hypohidrotic Ectodermal Dysplasia in Fleckvieh Cattle

**DOI:** 10.3390/genes15010008

**Published:** 2023-12-20

**Authors:** Sina Reinartz, Christine Weiß, Maike Heppelmann, Marion Hewicker-Trautwein, Maren Hellige, Laure Willen, Karsten Feige, Pascal Schneider, Ottmar Distl

**Affiliations:** 1 Institute for Animal Breeding and Genetics, University of Veterinary Medicine, 30559 Hannover, Germany; sina.reinartz@amedes-group.com; 2 Clinic for Swine, Ludwig-Maximilians-Universität München, 80539 Munich, Germany; christlwhite@icloud.com; 3 Clinic for Cattle, University of Veterinary Medicine, 30173 Hannover, Germany; maike.heppelmann@tiho-hannover.de; 4 Institute of Pathology, University of Veterinary Medicine, 30559 Hannover, Germany; marion.hewicker-trautwein@tiho-hannover.de; 5 Clinic for Horses, University of Veterinary Medicine, 30559 Hannover, Germany; maren.hellige@tiho-hannover.de (M.H.); karsten.feige@tiho-hannover.de (K.F.); 6 Department of Immunobiology, University of Lausanne, 1066 Epalinges, Switzerland; laure.willen@unil.ch (L.W.); pascal.schneider@unil.ch (P.S.)

**Keywords:** ectodysplasin A, Fleckvieh, missense mutation, collagen triple helix repeat domain

## Abstract

Mutations within the *ectodysplasin A (EDA)* gene have been associated with congenital hypotrichosis and anodontia (HAD/XHED) in humans, mice, dogs and cattle. We identified a three-generation family of Fleckvieh cattle with male calves exhibiting clinical and histopathological signs consistent with an X-linked recessive HAD (XHED). Whole genome and Sanger sequencing of cDNA showed a perfect association of the missense mutation g.85716041G>A (ss2019497443, rs1114816375) within the *EDA* gene with all three cases following an X-linked recessive inheritance, but normal *EDAR* and *EDARADD*. This mutation causes an exchange of glycine (G) with arginine (R) at amino acid position 227 (p.227G>R) in the second collagen triple helix repeat domain of EDA. The *EDA* variant was associated with a significant reduction and underdevelopment of hair follicles along with a reduced outgrowth of hairs, a complete loss of seromucous nasolabial and mucous tracheal and bronchial glands and a malformation of and reduction in number of teeth. Thermostability of EDA G227R was reduced, consistent with a relatively mild hair and tooth phenotype. However, incisors and canines were more severely affected in one of the calves, which correlated with the presence of a homozygous missense mutation of *RNF111* (g.51306765T>G), a putative candidate gene possibly associated with tooth number in *EDA*-deficient Fleckvieh calves.

## 1. Introduction

X-linked hypo- or anhidrotic ectodermal dysplasia (XHED/XAED/EDA/ED1) is an inherited congenital disease known in humans [1], mouse [2], cattle [3,4,5,6,7,8,9,10,11,12,13,14,15,16] and dogs [17]. Clinical signs in human include sparsely or absent hairs (hypo-/atrichosis), abnormally shaped or missing teeth (hypo-/anodontia) and a deficient development of eccrine glands (sweat glands, mucous glands in the respiratory tract, mammary glands) with the consequence of lethal hypo- or anhidrosis. Cattle with XHED show missing teeth in a variable extent, reduced number and underdeveloped hair follicles and dilated sweet glands [7,9,12,13,14] (Tables S1 and S2). In cattle, several mutations within the *ectodysplasin A (EDA)* gene had been reported (Figure S1). The *EDA* gene encodes the transmembrane protein ectodysplasin 1, which is involved in the early epithelial-mesenchymal interaction and the formation of hair follicles, mammary glands and tooth buds. In humans, for this gene, eight different but only two functional transcripts are known. The functional isoforms *EDA-A1* and *EDA-A2* bind to specific receptors encoded by the genes *EDAR* and *EDA2R* [18]. Variants at EDAR were linked to hair thickness and specific tooth morphology [18]. Recently, a frameshift mutation within *EDAR* (p.P161RfsX97) in Charolais calves with a common paternal ancestor was associated with generalized hypotrichosis and oligodontia in homozygous animals [19]. Molecular studies demonstrated that more than 300 genes are involved in tooth formation and development [20]. The most important candidate genes are *MSX1*, *PAX9*, *AXIN2*, *EDA*, *EDAR*, *EDARADD* and *WNT10A.* Dominant loss-of-function mutations in *MSX1, PAX9* and *AXIN2* had been implicated in familial forms of non-syndromic tooth agenesis [21,22,23]. The EDA–EDAR–EDARADD signaling pathway is involved in tooth growth and morphogenesis at different stages during tooth development, particularly determining tooth number and contributing to cusp formation [24,25]. Even additive effects of different mutations in these genes were suspected [26,27,28]. In EDA-heterozygous woman, the phenotype manifested itself in 10 to 30% of the cases [29], generally in milder forms than in hemizygous men [30]. In cattle, primarily XHED/XAED affected male calves were reported. Only very few XHED/XAED-heterozygous female cattle showed mild clinical signs [4,16]. A unique case of a Holstein female calf showed a severe phenotype similar to that of hemizygous males [31]. A large de novo chromosomal inversion disrupting both genes, *EDA* and *XIST*, may have caused the inactivation of the normal X chromosome and may have resulted in an abnormal EDA protein. In human, XHED/XAED was often associated with the absence or underdevelopment of the mammary glands which are of ectodermal origin as modified sweat glands [32]. In XHED/XAED-heterozygous woman, an insufficient milk production was determined in 80% of cases [33]. In transgenic mice, the overexpression of the *EDA-A1* isoform led to an increased number of teeth, hair follicles and mammary glands [34]. In cattle, the effects of *EDA* variants on glandular tissue had only been found on nasolabial [7], tracheal and bronchial glands [10]. On the X chromosome near the *EDA* gene at 87.2 Mb, a quantitative trait locus (QTL) for milk yield was detected in Chinese Holstein cattle [35]. The objectives of the present study were to characterize the course of the phenotypic development of XHED-affected calves from a Fleckvieh cattle family with a common female ancestor using clinical and histopathological examinations and to search for associated mutations with whole genome sequencing (WGS) data shared by all three cases. In addition, we searched WGS data for mutations affecting genes involved in teeth development because we observed marked differences in tooth agenesis among the three familial cases.

## 2. Materials and Methods 

### 2.1. Ethics Statement

All animal work was conducted according to national and international guidelines for animal welfare. The Lower Saxony state veterinary office at the Niedersächsisches Landesamt für Verbraucherschutz und Lebensmittelsicherheit, Oldenburg, Germany, was the responsible Institutional Animal Care and Use Committee (IACUC) for this study. This specific study had been approved by the IACUC of Lower Saxony, the state veterinary office Niedersächsisches Landesamt für Verbraucherschutz und Lebensmittelsicherheit, Oldenburg, Germany (registration number 33.42502-05-04A427 and 33.12-42502-04-15/2049).

### 2.2. Animals

**Figure 1:**
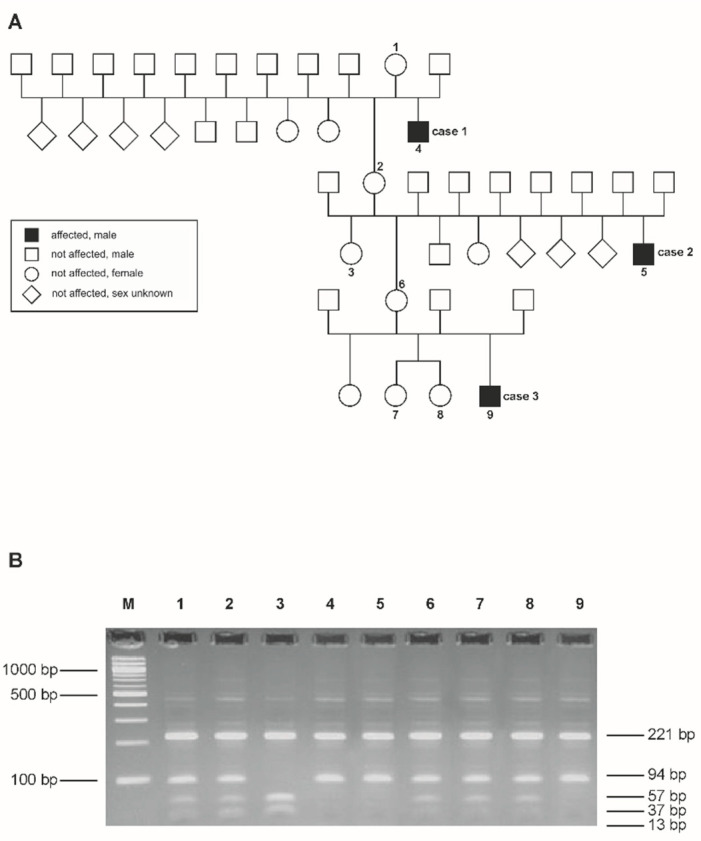
Pedigree of the Fleckvieh cattle family with the three affected male calves (cases 1–3) and the corresponding results from the PCR-RFLP analysis for the EDA:g.85716041G>A (ss2019497443, rs1114816375) variant. (**A**) Pedigree of the Fleckvieh cattle family used in this study. Samples from the numbered animals were available for the molecular genetic analyses. Phenotypic data from the other animals are based on veterinary examinations and the farmer’s records. (**B**) Agarose gel electrophoresis with the results of the PCR-RFLP analysis for the rs1114816375 variant. The lane designated M is a 100-bp ladder marker. The affected male animals (numbered 4, 5 and 9 on the agarose gel) had only the mutant allele with fragment lengths of 221, 94 and 13 base pairs. The maternal half-sister of case 2 (numbered 3 on the agarose gel) had only the wild-type allele with 221, 57, 37 and 13 base pair fragments. Dams of case 1 (numbered 1 on the agarose gel), case 2 (numbered 2 on the agarose gel), and case 3 (numbered 6 on the agarose gel) as well as two maternal half-siblings (numbered 7 and 8 on the agarose gel) of case 3 have both mutant and wild-type alleles, indicating that they are heterozygous.

According to the report of the farmer several years ago a male Fleckvieh calf with lack of a hair coating at certain body parts and defective teeth was born on a dairy farm in Bavaria, Germany, with approximately 40 dairy cows. In the following years, a further male Fleckvieh calf with similar conditions was born in this herd. These two calves developed normally until weaning, but after weaning, growth rates were poor. At the age of six months, both calves were slaughtered. Pedigree and clinical data of these two calves were not available. Further three purebred Fleckvieh male calves with this condition were born in March and September 2015 and February 2016. These three calves were brought at the age of six (case 1), five (case 2) and four (case 3) weeks to the Institute of Animal Breeding and Genetics of the University of Veterinary Medicine Hannover. Case 1 was the tenth calf of a Fleckvieh cow, case 2 was the eighth calf of a maternal half-sister of case 1 (daughter of the dam of case 1) and case 3 was the fourth calf of a maternal half-sister of case 2 (granddaughter of the dam of case 1) (Figure 1). The sires in this herd were exclusively artificial insemination Fleckvieh bulls. The relationships of the first two anecdotal cases with cases 1, 2 and 3 could not be established as no herdbook data were available for this herd. The head, the ears, the dorsal neck and the back of case 1 were hairless at birth. The backside of the calf was more hairy. The first two anecdotal cases had a similar appearance according to the report of the farmer. Case 2 was hairless at the head, ear pinnae, metacarpi and metatarsi, and case 3 was hairless only on the head and the ear pinnae at birth. The development of cases 1, 2 and 3 were monitored, including a regular recording of the body weight. At the age of seven weeks, and a second time at ten months, a dorso-lateral and latero-lateral radiographic examination of the skull of case 1 was performed using the Gierth X-Ray international system (Gierth Hf400V, Gierth X-Ray international, Riesa, Germany) with 55 kV and 22 mAS, focus-film distance of 1 m with the animal positioned in lateral recumbency under xylazin-sedation (0.1 mg/kg). For case 2, the same radiographic examination of the skull was carried out at the age of four months. Case 1 had an infection of the umbilical cord, which was surgically extirpated at the age of ten weeks. During the course of this operation, skin punch samples were taken for histopathological examinations and tissue samples were collected for RNA isolation. EDTA-blood samples were sampled from all cattle of this farm. Samples of cattle from breeds other than Fleckvieh were derived from our bio-bank for diagnostic purposes (Table S3). 

### 2.3. Histopathological Examination

Two skin punch biopsies of 6 mm diameter were collected from the flank (normal-appearing coat but with an abnormal silky texture of the hairs) and neck (hairless at birth and later in life with sparse and shorter hair) of case 1 at the age of ten weeks and for a second time at the age of ten months. Two skin punch biopsies were also collected from the flank (normal-appearing coat but with an abnormal silky texture of the hairs) and head (hairless at birth) of case 2 at the fourth month of life and of case 3 at the age of ten weeks. In addition, biopsies from the muzzle were obtained from both animals at the age of ten weeks and four months, respectively. After the slaughtering of case 1 at the age of 17.5 months and case 2 at 11.5 months, further skin samples from the head, ear, tail tassel and legs of both cases were histologically examined and hair follicles per mm^2^ were counted. In addition, the tracheal, bronchial, salivary, Meibomian glands, claws, testicles and the teats were studied for their presence and morphology. All samples were fixed in 10% neutral formalin and embedded in paraffin wax. From each paraffin-embedded skin sample sections (3–4 µm) were cut, stained with hematoxylin and eosin (H&E) and examined with a light microscope. On additional skin sections, the periodic acid-Schiff (PAS) reaction was performed. For the counting of hair follicles in H&E-stained skin sections of case 1, which were taken at ages of ten weeks and ten months, a binocular microscope, a 10× objective and a 10× ocular with a square eyepiece graticule (10 × 10 squares with a total area of 100,000 µm^2^) were used. On two skin sections in each of the two biopsies, the hair follicles were counted in 8 areas (flank) and in 9 areas (spinal), respectively. The results, i.e., the number of hair follicles, were calculated and expressed as follicles per 1 mm^2^.

### 2.4. Cytogenetics

For the cytogenetic presentation of the chromosomes of cases 1–3, a heparinized blood sample was collected from the vena jugularis. The blood sample was incubated with medium, antibiotics and phytohaemagglutinin for 72 h at 38 °C and in 5% CO_2_/air. Colcemid was added at a final concentration of 15 mg mL^−1^ for 1.5 h before harvesting. Chromosome preparation followed standard methods [36] for hypotonic treatment with 0.075 M KCl and fixation with 3:1 methanol/glacial acetic acid. Air-dried slides were stained with giemsa and giemsa–trypsin–giemsa (GTG) banding was performed. Metaphase chromosomes were photographed with a computer-controlled CCD camera and processed with IPLAB 2.3 software.

### 2.5. RNA and DNA Extraction

Tissue samples of case 1 from the umbilical region were collected during a surgical intervention due to an umbilical infection. Hair samples of the tail were used for case 2. Samples were immediately stored in liquid nitrogen. TRIZOL reagent (Life Technologies, Heidelberg, Germany) and Qiagen RNeasy Lipid Tissue Kit (Qiagen, Hilden, Germany) were used to extract total RNA from tissue samples according to manufacturer’s protocol. Aliquots of 1 µg total RNA were reverse transcribed into cDNA using 20 pmol (T) 24 V primer and Omniscript Reverse Transcriptase (Qiagen) in 20 µL reactions. Genomic DNA was extracted using 500 µL EDTA-blood and a standard ethanol fraction [37].

### 2.6. EDA, EDA2R and WNT10A Sequencing

Genomic primer pairs were designed to amplify exons 1 and 9 of *EDA* (Table S4). These primer pairs had a product size of 718 bp and 794 bp. All exons with their adjacent introns or UTRs of the *EDA2R* and *WNT10A* gene were amplified and sequenced. PCR was performed in 22 μL reaction volumes containing 2 μL of the cDNA or genomic DNA, 70 μM deoxyribonucleoside triphosphates, 5 pmol of each primer and 3.5 U of *Taq* polymerase in the reaction buffer supplied by the manufacturer (Qiagen). After a 5 min initial denaturation at 94 °C, 36 cycles of 30 s at 94 °C, 1 min at 60 °C and 45 s at 72 °C were performed in a T Professional Thermocycler (Whatman Biometra, Goettingen, Germany). All amplicons from the affected and a control animal were sequenced with forward and reverse sequencing primers on a Genetic Analyzer 3500 (Applied Biosystems by Life Technologies, Darmstadt, Germany). Sequences were analyzed with Sequencher 4.8 (Genes Codes, Ann Arbor, MI, USA). Sequences were screened for mutations using the bovine reference sequence from NCBI (ARS-UCD2.0, GCF_002263795.3) and Ensembl (ARS-UCD1.2, GCA_002263795.2).

### 2.7. Whole Genome Sequencing

DNA samples from all three cases were prepared for sequencing using the NEBNext Ultra II DNA Library Prep Kit for Illumina for high quality libraries (NEB, New England BioLabs, Ipswich, MA, USA). Size selection and indexing was quality controlled on an Agilent 2100 Bioanalyzer system with a High Sensitivity DNA kit (Agilent Technologies, Santa Clara, CA, USA). Finally, indexed libraries were sequenced on an Illumina NextSeq 500 for 300 cycles in 2 × 150 paired-end modes. After quality control of fastq files using fastqc 0.11.3 (http://www.bioinformatics.babraham.ac.uk/projects/fastqc/, accessed on 27 July 2023), reads were mapped to the ARS-UCD1.2 bovine reference genome using BWA 0.7.12 [38], were converted into binary format and underwent variant calling using SAMtools 1.3.1 [39], Picard tools (http://picard.sourceforge.net, version 2.3.0, accessed on 27 July 2023) and GATK 3.5 [40]. Further variant effect prediction and classification of effects was carried out using SNPEff version 4.1 b [41]. In addition, variant calling and bioinformatic analysis included 15 whole-genome sequences of different cattle breeds (Fleckvieh, Holstein, Galloway, Limousin cattle) retrieved from the Sequence Read Archive (NCBI) as controls. Genetic variants submitted to the dbSNP database were used as further controls. All fastq data can be obtained through the NCBI Sequence Read Archive (http://www.ncbi.nlm.nih.gov/sra, accessed on 27 October 2016), BioProject PRJNA350739.

### 2.8. Validation

A PCR-restriction fragment length polymorphism (PCR-RFLP) was developed for the EDA:g.85716041G>A (rs1114816375) variant. Primer pairs, amplicon size in base pairs (bp), annealing temperature, restriction enzyme and incubation temperature are given in Table S5.

### 2.9. RNA Expression, and Production of Recombinant EDA1

The expression analysis of cDNA was performed using the primer pairs as previously described [8]. Expression plasmids for full-length human EDA1 with mutation N163S plus or minus G227R were constructed according to standard molecular biology techniques. They were transfected with polyethyleneimine in 293T cells grown in 10 cm plates according to the protocol of Tom et al. [42]. The day after, cells were washed and grown for 4 days in 8 mL of serum-free OptiMEM medium, after which cells and conditioned supernatants were harvested. The cell pellet was sonicated in 300 µL of sample buffer. Amounts of 0.33 µL of cell supernatant and 0.66 µL of cell pellet were heated for 3 min at 70 °C in 30 µL of reducing (dithiothreitol) SDS-PAGE sample buffer and analyzed by 12% acrylamide SDS-PAGE, followed by Western blot with Renzo-2 anti-EDA monoclonal antibody (Enzo Life Sciences, Farmingdale, NY, USA, ALX-522-038). 

### 2.10. Cell-Based EDA1 Activity Assay

Recombinant EDA1 in conditioned cell supernatants were concentrated 20-fold using a centrifugal device with a 30 kDa cutoff membrane. Jurkat JOM2 cells expressing the chimeric receptor EDAR:Fas (JOM2-EDAR:Fas-2199 clone 23) were exposed for 16 h to titrated amounts of concentrated supernatants containing naturally processed EDA1, after which time cell viability was measured with the phenazine methosulfate/(3-(4,5-dimethylthiazol-2-yl)-5-(3-carboxymethoxyphenyl)-2-(4-sulfophenyl)-2H-tetrazolium (PMS/MTS) test [43]. 

## 3. Results

### 3.1. Phenotype

**Figure 2:**
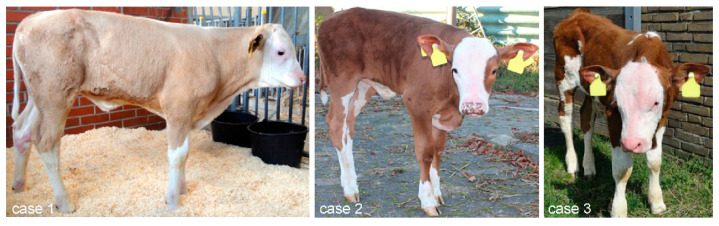
Cases 1–3 at the age of six weeks with almost identical development of the coat. A thin fluff of hairs covered the heads, the necks, the backs and the feet of cases 1–3. The sparse coat is most obvious on the bridge of the nose and around the eyes. The outer edges of the outer sides and the inner sides of the ear pinnae were hairless. The rest of the body was covered with longer hairs. The hair on the whole body appeared very fine, soft and silk-like. On the tip of the tail, hair began to grow but the tail tassel only showed 1/3 of the normal length. The muzzle had a rough and dry surface. The expressivity of the underlying *EDA* mutation thus appears to be rather homogeneous with regard to the coat phenotypes.

In a Bavarian dairy farm, three dams gave birth to male calves with clinical signs consistent with EDA. The calves were born in March 2015 (case 1), September 2015 (case 2) and February 2016 (case 3) and reared at the barns of our institute until September 2016. All three calves had no visible hairs at birth on the heads, ears, necks, backs, feet and tips of the tails (Figure S2). At the age of six weeks, these body parts were covered by a thin fluff of hairs with the exception of the outer and inner sides of both ear pinnae and especially around the eyes. On the bridge of the nose, hairs were very sparse (Figure 2). The hairs on the whole body appeared very fine, soft and silk-like. Excoriations on mechanically exposed areas were seen on both heels bumps, above the claws and on the root tail only at this time of life.

On the tip of the tail, hairs began to grow but the tail tassel was only 1/3 the normal length at all ages up to 1.5 years of age (Figure S3). At the age of 7 months, all parts of the body of cases 1, 2 and 3 were covered with normal looking, fine, soft and curly hairs, except for the ear pinnae, as well as the dorsal area from the neck to the shoulders (Figure S4). The outer edges of the outside and inside of the ear pinnae were still hairless. The dorsal part between neck and shoulders was covered with shorter and a less dense coat than the other areas of the body. The muzzle was dry and rough in all three cases. The horns and the claw horn developed normally (Figure S5). The coat of case 1 lost curliness at the age of 1.5 years (Figure S6). The coat of case 2 retained curly hairs until the age of 11 months but in an attenuated fashion as compared to the seventh month of life. 

**Figure 3:**
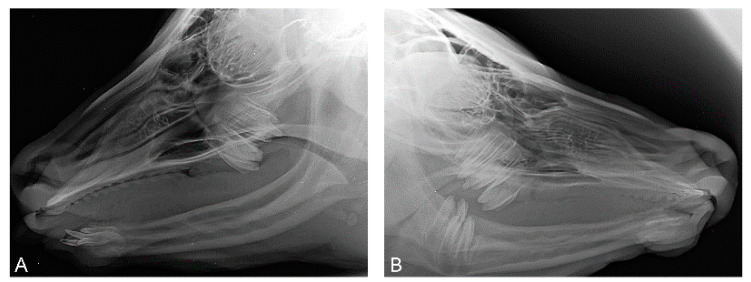
Radiographs of the head of case 1 at the age of seven weeks (**A**) and of the head of case 2 at the age of four months (**B**). (**A**) Latero-lateral view. Four maxillary molars, two on each side, were visible. Apart from the two erupted incisors, two shorter, not erupted, also pointed canines were seen. A total of eight teeth were present. (**B**) Latero-lateral view. Six molars, three on each side on both sides, were visible. Incisors and canines were absent.

At the age of 7 weeks, case 1 had developed one lateral incisor (I3) on each side (Figure 3).

**Figure 4:**
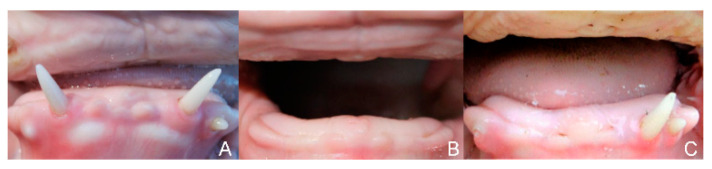
Development of incisors and canines of the three affected calves, each at the age of seven months, with different expressivity of the tooth phenotypes. (**A**) Two malformed and conical teeth with a length of 1.3 cm were visible protruding from the gums of case 1. At the age of seven months, one canine broke through the gums on the left side and the second canine was covered by a gum cap. (**B**) No incisors or canines developed in case 2 throughout its life. (**C**) At the age of 7 months, the canines broke through the gums on both sides. The right-sided canine stayed short. The development stage of the canines resembled those of case 1.

These incisors (case 1) were visible as rice grain-like structures at the mandible. By the tenth week, conic and up to 1.3 cm long teeth had grown. At the age of 7 months, the canine on the left side broke through the gums and the canine on the right was covered by a gum cap (Figure 4). In case 2, neither incisors nor canines developed up to the age of 11.5 months. In case 3, at the age of ten weeks, a spiky incisor had broken through the gum on the left side.

At the age of 7 weeks, the presence of each two molars on each side were seen on the X-rays of case 1, but mandibular premolars were absent. Case 2 did not show incisors or canines at clinical examination or on the X-rays at the age of 4 months. Using X-rays, two maxillary molars on each side and the mandibular molars on both sides of case 2 were observed. In case 3, a spiky incisor (I3) broke through the gums on the left side at the age of ten weeks. At the age of four months of case 3, computed tomography of the head demonstrated the presence of canines on both sides of the mandible, two molars on each side of the maxilla and one molar on both sides of the mandible. At the age of 7 months, the canines broke through the gums on both sides. The right-sided canine stayed short. The development stages of the canines resembled those of case 1. No further development of teeth was observed throughout the rest of the lives of all three cases. 

Case 1 reached an appropriate weight in the second month of life, which is within the range of female Fleckvieh calves in the first month of life (Table S6). This initially poorer development of the animal strongly improved from the 2nd to the 4.5th month of life, to the point that its weight in the 4.5th month was identical with those reported for female Fleckvieh calves [44]. The affected calf had a daily weight gain of 1160 g between the 2nd and 4.5th month of life. Such daily gains were also observed for case 1 between the tenth and twelfth month of life. Between the fourth and sixth month of life the daily weight gain decreased to 548 g. Case 2 weighed 30 kg more in the second month of life than case 1 but it had daily weight gains of only 600 to 700 g until the sixth month of life.

### 3.2. Histopathological Findings

H&E stained sections of skin biopsies at the age of 10 weeks from cases 1, 2 and 3 showed most hair follicles in the telogen phase with normal hair bulbs. Hair follicles in the telogen phase had either hairs or were hairless (Figure S7). In hairless follicles, slight hyperkeratosis was often found. Sweat and sebaceous glands were present in all the examined skin samples and were physiologically developed. The sweat glands had a diffused, moderate dilation of lumina, which contained PAS positive material indicative for active secretion. In the superficial dermis, a slight perivascular infiltration with lymphocytes, macrophages and eosinophilic granulocytes was seen. The epidermis, sebaceous glands, dermal collagen and arrector pili muscles appeared normally developed. In skin samples from the flank and neck of case 1, 21.88 and 22.77 hair follicles per mm^2^, respectively, were counted at the age of 10 weeks. A repeated biopsy for case 1 at the age of 10 months showed 10.42 (flank) and 14.24 (neck) hair follicles per mm^2^. Underdeveloped hair follicles and channels (hair infundibulum with rudiments and broken hair) were particularly striking at the neck in both biopsies taken at the age of 10 weeks and 10 months in contrast to the flank region (Figure S7). In skin biopsies from case 2 of the flank and head, 11.50 and 22.22 hair follicles per mm^2^, respectively, were counted at the age of 4 months. Case 1 and 2 were slaughtered at the age of 17.5 and 11.5 months of age, and after slaughtering, further tissue specimens were collected for investigation. In the skin of the ear tips, a lack of hair follicles (case 1), a focal lack of hair follicles (case 2), and underdeveloped hair follicles and a slight follicular hyperkeratosis were detected. In cases 1 and 2, 1.34 and 2.50 hair follicles per mm^2^ in tissue samples from the ear tips were counted. The skin in the tail tassel was hairless with dilated follicles, and counts revealed 3.54 and 7.32 hair follicles per mm^2^ in cased 1 and 2. The numbers of hair follicles per mm^2^ in the skin of the heads of cases 1 and 2 were 9.0 and 5.63. The hair follicles did not contain hairs and had dilated sweat glands with a slight follicular hyperkeratosis. The skin on the legs was empty, dilated and displayed underdeveloped hair follicles with slight follicular hyperkeratosis with 11.41 and 11.25 hair follicles per mm^2^ in case 1 and 2. 

**Figure 5:**
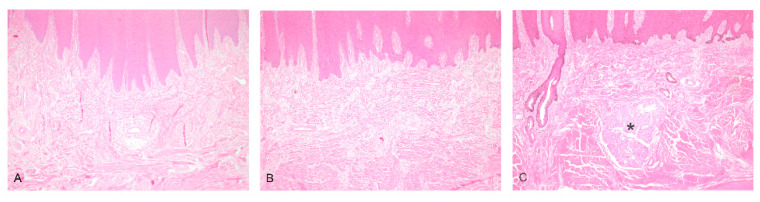
Sections from the muzzles of cases 1 (**A**) and 2 (**B**), in which the nasolabial glands are absent, and from a normal control animal with nasolabial glands (asterisk) (**C**). H&E stain, ×40.

In biopsies from the muzzles of cases 1 and 2 at the ages of 10 and 4 months, the absence of the nasolabial glands was evident (Figure 5).

After slaughter, histopathological sections revealed that the seromucous nasolabial, mucous tracheal and bronchial glands were completely lacking in cases 1 and 2, whereas the salivary glands and the Meibomian glands were normally developed. Case 2 had a slight multifocal suppurative bronchiolitis and alveolitis. The corium and the horns of the claws, the testicles and the teats were normally developed in both cases.

### 3.3. Pedigree Analysis and Cytogenetics

The pedigree for the three cases is consistent with a monogenic X-chromosomal recessive trait (Figure 1). Common ancestors of the three male affected animals were only found in the maternal line. The clinical examination of dams and half-siblings provided no evidence of abnormal coat hair and teeth development.

We were not able to detect abnormal chromosomes in the karyotype of case 1 (2*n* = 60, XY) (Figure S8). For the other two affected calves, we were also able to confirm that the karyotypes were not abnormal.

### 3.4. Whole Genome Sequence and Mutation Analysis

**Figure 6:**
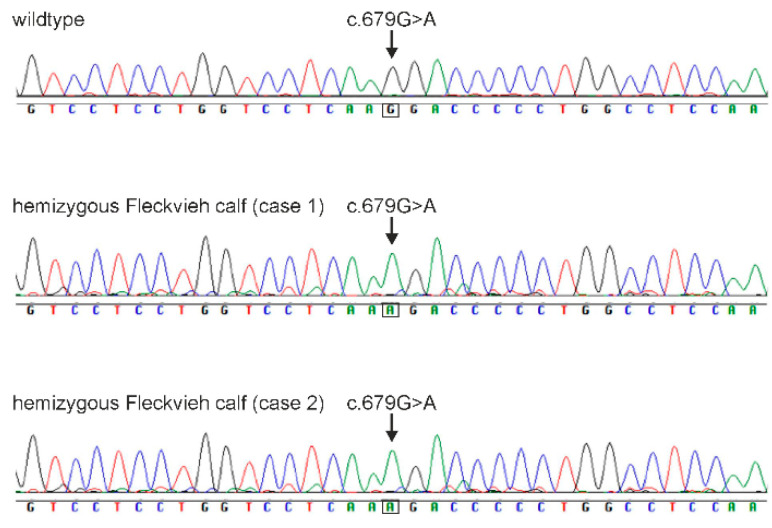
Validation of the *EDA*-associated mutation using Sanger sequence analysis (chromatogram) of exon 4 from a wildtype Fleckvieh and two XHED-affected male Fleckvieh calves (cases 1 and 2). The arrow indicates the position of the c.679G>A mutation within the cDNA sequence of *EDA*.

We searched WGS data of all three cases for private mutations with special regards to the genes of the *EDA–EDAR–EDARADD* pathway including *EDAR*, *EDARADD*, *TRAF6*, *TKK1*, *TAK1*, *TAK2*, *IKK1*, *IKK2*, *NEMO*, *JNK*, *NFKB* and *IKB*. In addition, the genes *WNT10A*, *WNT10B*, *WNT6*, *MSX1*, *AINX2* and *PAX9*, due to their implication in development of incisors and premolars, were included. Using WGS data, we were able to retrieve a total of three homozygous or hemizygous variants private for the three cases including the EDA:g.85716041G>A mutation. The remaining two variants were located in pseudogenes (ENSBTAG00000046816, OR51G1-201 and ENSBTAG00000039185) on BTA15. In all candidate genes other than *EDA*, we were not able to detect mutations influencing coding sequence in any of the three cases or harboring homozygous or hemizygous private mutant genotypes. Both X-linked genes possibly involved in XHED, *EDA* and *EDA2R*, as well as *WNT10A* due to its involvement in teeth development, were sequenced using cDNA and Sanger sequencing. The sequence analysis of the cDNA of the *EDA* gene for case 1 and 2 confirmed the *EDA*-associated missense mutation within exon 4 (g.85716041G>A, c.679G>A, ss2019497443, rs1114816375) (Figure 6). In addition, we searched WGS data for homozygous mutant genotypes private to case 2 and not present in cases 1 and 3. Case 2 did not develop incisors and canines like cases 1 and 3. We found 12 variants with low, moderate or high impacts on protein structure using SNPEff, of which three mutations were located in annotated genes. These genes were *RNF111* on BTA10 (g.51306765A>C, c.1895A>C), *ZFAND4* on BTA28 (g.44551736insCTAGT, c.184+3_184+4insCTAGT) and *OPCML* on BTA29 (g.35069027C>T, c.801C>T). A deleterious effect with high confidence was predicted for the bovine RNF111:g.51306765A>C mutation using SIFT (0.06).

The missense mutation within *EDA* causes an exchange of glycine (G) with arginine (R) at amino acid position 227 (p.227G>R). A probably damaging effect on the resulting protein was predicted using PolyPhen-2 [45] with a score of 99.7%. A deleterious effect with low confidence was predicted by SIFT. According to the bovine reference genome assembly ARS-UCD2.0 and GCF_002263795.3, this missense mutation is localized in the collagen triple helix repeat domain of the EDA protein. Further mutations in the cDNA and DNA sequences of the genes *EDA2R* and *WNT10A* were not found.

### 3.5. Validation

In order to validate the EDA-associated missense mutation in a larger number of animals, a RFLP (restriction fragment length polymorphism) was developed using the restriction enzyme NlalV (Figure 1). Cases 1, 2 and 3 were shown as hemizygous for the mutated allele A, while the dams of all three cases and two maternal half-sisters (twins) of case 3 were heterozygous for the *EDA* rs1114816375 variant. A maternal half-sister of case 2 was homozygous for the wildtype. Furthermore, the mutation was genotyped in all other available animals of the Fleckvieh herd where the cases were ascertained, and in addition, in 300 unaffected animals from different farms and breeds (Table S3). All controls were homozygous for the wildtype. We found a perfect association of the EDA:g.85716041G>A variant with the XHED-affected phenotypes and obligatory carriers. An X-linked recessive inheritance was confirmed based on the genotypes for the EDA variant and the clinical examination of the dams and maternal half-siblings of the XHED-affected male calves.

### 3.6. RNA and Protein Expression

Amplicons of exon 1 to exon 9 of the *EDA* gene of the cases 1, 2 and the control animal had sizes of 818 bp and did not differ in size from each other (Figure S9).

**Figure 7:**
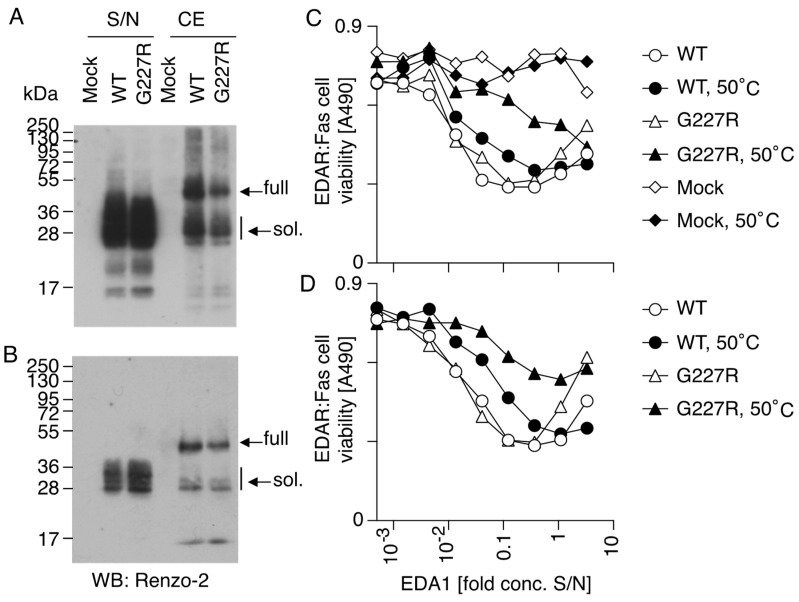
Western blot analysis and activity of naturally processed recombinant EDA1, WT or G227R. Expression plasmids for full-length EDA1, with or without the mutation G227R, or an empty plasmid (Mock), were transfected in 293T cells. (**A**) Western blot analysis of cell extracts (CE) or conditioned cell supernatants (S/N) under reducing conditions using Renzo-2, a monoclonal anti-EDA antibody. (**B**) Same as panel A, with an independent transfection. (**C**) Cell supernatants pre-incubated or not for 60 h at 50 °C were tested at the indicated concentrations for their activity on EDAR:Fas reporter cells, and cell viability was monitored. Decreased activity at higher ligand concentration is usual in this type of assay and probably reflects inadequate ligand-induced receptor clustering at higher ligand concentrations. (**D**) Same as panel C, with supernatants of an independent transfection. Experiments in panels C and D were performed twice with similar results.

Next, we investigated the consequence of mutation G227R on the expression, processing, receptor-binding, activity and stability of the EDA1 protein. Bovine and human EDA differ by a single amino acid (N163 in human, S163 in cow); this residue is located between the furin processing site and the collagen domain of EDA, away from the domain that can bind and engage EDAR. Thus, expression plasmids of human EDA1 with mutation N163S, with or without the pathogenic mutation G227R, were prepared, so that a soluble EDA1 with the bovine sequence would be released from transfected cells after proteolytic processing. Two main EDA bands were detected via Western blot in cell extracts, corresponding to full-length EDA1 and processed EDA1, while only processed EDA was released into supernatants. Sizes of these bands were a bit higher than the predicted sizes of 41.2 and 24.2 kDa for full and processed EDA1, because of N-glycosylations [46]. Mutation G227R did not obviously affect the expression, processing and solubility of the released protein in two independent transfections (Figure 7A,B). Unexpectedly, EDA1 WT and G227R had similar activities when monitored with EDAR:Fas reporter cells. In these cells, the binding of EDA1 to EDAR does not stimulate the natural EDAR signaling pathway but instead promotes cell death via the intracellular domain of the death receptor Fas. As Fas signaling is exquisitely sensitive to the aggregation status of the ligand, it was anticipated that a mutation in the collagen domain would impair the multimerization of EDA1 and decrease or abolish its activity [47], but this was not the case (Figure 7C,D, white circles and triangles). However, when proteins were pre-incubated for 60 h at 50 °C, EDA1 G227R lost more activity than EDA1 WT, indicating that the mutation probably decreases thermostability (Figure 7C,D, black circles and triangles).

## 4. Discussion

A previously not yet identified missense mutation within the *EDA* gene was identified in a German Fleckvieh family, which segregated for X-linked HED. The EDA-associated mutation was perfectly segregating between the cases and their unaffected dams in a three-generation pedigree. All male animals with the susceptible hemizygous *EDA* genotype A expressed the XHED phenotype; thus, penetrance was 100%. Heterozygous mutant females did not show any signs consistent with the XHED phenotype, resulting in a penetrance of 0% for females with a heterozygous *EDA*-mutated genotype. The hair follicle development and underdevelopment of coat hairs were nearly but not completely identical among the three cases in the present study. Therefore, we propose that the expressivity with regard to coat hairs may be slightly variable despite the same mutated *EDA* genotype. The head, ears, dorsal neck and back of case 1; the head, ear pinnae, metacarpi and metatarsi of case 2; and the head and the ear pinnae of case 3 were completely hairless at birth, whereas the other body parts were covered by a normal-looking coat with silk-like hairs. In all three cases, the tail tassel showed hairs of only 1/3 of the normal length. Horns and claws developed normally. In agreement with the present report, the degree of hypotrichosis was not identical in four affected Holstein calves [7]. In contrast to previous reports [3,5,7,14,15,48] where the entire body of affected calves was hairless or only a few tufts of hairs were seen, the present cases had fewer body parts with missing coat at birth.

The increasing activity of hair follicles was observed in all three cases. At the age of six weeks, the affected body parts were covered by a thin fluff of hair. Especially around the eyes and on the bridge of the nose, coat hair was very sparse. In the fifth month of life, only the insides and the outer edges of the outside of the ear pinnae appeared symmetrically hairless, and the tail tassel remained at 1/3 of the normal length. The development of the coat hair was also observed in previously described cases at later stages of life [3,5,7,9,48]. The face, neck, back, tail, ears and area around the eyes are body parts which were previously reported as hairless in all cases [3,4,7,9,10,13,16,48]. But with the increasing age of these animals, it became obvious that only certain areas remained completely hairless. These body parts were the pinnae of the ears and the areas around the eyes [10,13]. In previous reports, the phenotypic traits of the affected animals were recorded at only one time point. Animals described only within the first few days or months of life exhibited an extremely sparse hair [7,9,12,14,15,16]. Animals with a slightly denser covering of hair were an eleven-month-old Swiss Fleckvieh heifer with a fine, downy coat [5]; a two-year-old German Holstein bull [10] with only hairless regions on the head, eyes and ear pinnae; and a seven-month-old animal from the Danish Red Holstein family [13], which showed a general short-haired coat and hairlessness around the eyes and the margins of the pinnae. We may assume that had the animals been examined at a later stage in life, they would have probably exhibited a more pronounced coat like the cases described here. In summary, the development of the coat seems to be delayed to a variable extent in XHED-affected cattle partly due to delayed development and partly due to the degeneration of hair follicles, particularly in the area around the eyes and outer edges of the ear pinnae.

In agreement with previous reports, we demonstrated a reduced hair follicle density [5,7,9,13], the dilation of the sweat glands [5,9,13], a higher proportion of hair follicles in the telogen phase [5,12,13,14] and perivascular dermatitis [13]. The number of hair follicles in normal cattle were at 57.6 ± 2.0 per mm^2^ in an one-day-old Jersey calf [49] and 30–60 per mm^2^ in an eight-month-old Norman cattle [50], whereas the counts of hair follicles from different body areas in the present study at different ages were at 1.3–22.8 hair follicles per mm^2^. These results show that case 1 and case 2 exhibited a greatly reduced follicle density in the areas with a visually normal coat as well as in the body parts with thin hairs on the parts which were hairless at birth. Upon the later histological examination of the flank and neck of case 1 at the age of ten weeks and ten months, underdeveloped hair follicles and channels (hair infundibulum with rudiments and broken hair) were found, particularly in the neck in contrast to the flank region. Accordingly, it was found that the hair follicles were reduced over the entire body, but that more hair follicles were underdeveloped in the areas that were hairless at birth. Based on biopsies taken at 10 weeks and 10 months of age, we observed a reduction in the number of hair follicles in case 1 at a later age. These results support the assumption that the hair follicle density declines with age. Further confirmation may be seen by the observation that in case 1, fewer hair follicles were counted in tissues when compared those from the same body areas of case 2. In case 2, the follicle density of the head also decreased from the 4th to the 11.5th month of life. Furthermore, a slight follicular hyperkeratosis was noticed in hairless follicles. 

A striking sign of XHED is a very dry muzzle and a high frequency of mouth moistening with the tongue, which is consistent with previous reports in Holstein cattle [7,9,10]. The absence of seromucous glands in the muzzle [7,9,13] and glandular structures in the trachea and bronchi [9,10,13] was demonstrated in XHED-affected Holstein cattle. The XHED-affected Fleckvieh calves from this study also had permanent dry muzzles, the absence of seromucous glands in their muzzles and the absence of mucous tracheal and bronchial glands. 

In contrast to all previous reports of *EDA*-deficiency in cattle, where incisors and canine were invariably absent, the development of incisors within the first weeks of life was observed in 2/3 cases of the present study, suggesting that the mutation only attenuates the activity of the protein, which is in line with our in vitro analysis of EDA1 G227R showing normal processing and receptor binding but reduced stability. In case 1, in the sixth week of life, the two rice grain-like incisors became visible, and by the tenth week of life, they had grown into two sharp, 1.3 cm long teeth. One canine broke through the gums at the age of seven months while the second canine remained within the gums. In case 2, no incisors developed, whereas in case 3, a spiky incisor developed on the left side and the canines later broke through the gums on both sides, while the right-side teeth stayed very short. The development stage of the canines resembled those of case 1. All previously described XHED-affected calves lacked incisors, except for a Swiss Fleckvieh heifer [5] and a Danish Red Holstein calf [13]. The Swiss Fleckvieh heifer had one spiky incisor [5]. The two incisors of the one-week-old Danish Red Holstein calf did not break through the gums [13]. Similarly, a different number of molars were found in full siblings [48], maternal half-siblings [7] or in related animals [13]. In the previous cases, premolars and molars in the mandible were almost completely absent, and in the maxilla, anterior premolars and molars were predominantly absent. In humans, central incisors, lateral incisors and canines of maxilla and mandible, with the possibility of persistence of maxillary and mandibular first permanent molars, suggest the presence of an *EDA* mutation [51]. In particular, the development of anterior dentition may be damaged in humans due to the size of the ectodermal signaling centers regulated by EDA signaling [52,53]. Tooth number, tooth cusp number and shape are under the control of Eda/Edar signaling in mice, but the coordination of signaling is controlled by different modulating mechanisms. Low Edar signaling thus results in fewer teeth with reduced and lower cusps. High Edar signaling is associated with ectopic distal teeth and the loss of the most proximal teeth and fine cusps in the first and second molar [24]. In patients with *MSX1*-associated mutations, especially the development of premolars was impacted [54] and heterozygous mutations in *PAX9* affected permanent molars [55,56]. Biallelic *WNT10A* mutations affected all tooth types in humans [56]. A severe mixed pattern of tooth agenesis resulted from autosomal dominant mutations in exon 7 of *AXIN2* [23]. These mutations were either excluded or do not correspond to the phenotype or transmission pattern of affected calves in this study. The hair normalized in the course of life in the three cases described here but the teeth remained severely damaged. This would fit with the hypothesis that higher thresholds for EDA/EDAR signaling are required in tooth development than in other EDA-dependent structures [57]. The development of teeth, especially of incisors and premolars, requires the highest dosage of EDA signaling. In an experiment, dog pups with the XHED syndrome were injected postnatally with recombinant EDA1. A relatively higher or repeated dosage of recombinant EDA1 was required for the development of permanent teeth [57]. The crown morphology of the incisors in the calves described here was conical to taper. This shape is a characteristic phenomenon for mutations in the *EDA* gene in humans [58]. In the Fleckvieh calves, the number of missing teeth was variable. Case 1 had eight, case 2 had six and case 3 had nine teeth present, compared to twenty and thirty-two teeth in age-matched normal calves and cows, respectively. In humans, a broad range of intra- and interfamilial variation in the severity of the HED syndrome, including number of missing teeth and shape of teeth, have also been detected [58,59]. In non-syndromic tooth agenesis, tooth number is also heterogeneous. A possible explanation may be a variable expression of a single shared genetic variant in the presence of sibling-specific sets of multiple other variants, each with weak individual effects, in other “modifier” genes that underlie the phenotypic variation [60].

The initial poorer weight gain of case 1 strongly improved from the 2nd to the 4.5th month of life, so that its weight in the 4.5 month was identical with those of normal Fleckvieh calves [44]. The daily weight gain of 1160 g between the 2nd and 4.5th month of life nearly reached a daily gain of 1300 g, which is in the normal range for fattening bulls through intensive fattening [61]. Such daily weight gains were observed again between the tenth and twelfth month of life. Between the fourth and sixth month of life, the daily weight gain decreased to 548 g. This was little more than half of the daily weight gain recorded in male Fleckvieh [62]. Case 2 had increasing weight gains in the first two months of life, but daily weight gains were only 600 to 700 g until the sixth month of life. These variable weight gains reflected the difficulty of feeding and caring for XHED-affected animals. The present cases were fed milk, cobs and pelleted concentrates, and in addition, had to be separated from other animals during feeding. Due to these problems with nutrition, farmers are not willing to keep XHED-affected animals for milk production or fattening. These figures also show that XHED-affected animals were able to reach similar daily weight gains with an appropriate feed supply, similarly to Fleckvieh bulls without such a defect. 

The EDA:g.85716041G>A mutation was in perfect association with all three XHED-affected Fleckvieh calves examined here and was consistent with an X-linked recessive inheritance. In agreement with all previously described XHED cases in cattle, mutations in the *EDA* gene were found to be associated with this condition. The EDA:g.85716041G>A mutation is located within exon 4, which belongs to the collagen triple helix repeat domain of the EDA protein. This domain may serve for the multimerization of EDA trimers to increase activity. In any case, it is important for the proper function of EDA in humans [63,64]. EDA1 is produced as a type 2 transmembrane protein of 391 amino acids (C-terminus outside the cell), which is proteolytically processed (between amino acids 159 and 160) at a furin consensus site to release the C-terminal portion, consisting of a roughly globular, trimeric TNF homology domain (amino acids 245–391) with three binding sites for the receptor EDAR; and a bi-partite collagen domain in which the first part (amino acid residues 180–210) is believed to form a bundle of collagen triple helices and the second part (amino acid residues 215–236) links the bundle to the individual receptor-binding trimeric globular domains [47]. Human mutation G207R in the first part of the collagen domain functionally destroyed the multimerization potential of the collagen domain, as tested in a setting of EDA1:FasL fusion proteins [47]. In contrast, the present mutation G227R in the second part of the collagen domain did not affect the activity of recombinant EDA1 but only decreased thermostability, suggesting that mutations in the first portion of the collagen domain might be more detrimental for the activity of EDA than mutations in the second portion. It would be interesting to compare mutations G207R and G227R side by side in the same experimental system to challenge this hypothesis.

The more severe tooth phenotype of case 2 compared to cases 1 and 3 might be due to the contribution of another gene or other genes in addition to *EDA*. Indeed, *OR51G1* encoding an olfactory receptor was proposed to potentially impact HAD, which may be possible as olfactory receptors perform diverse functions in cutaneous cells and wound healing [65,66]. The search for a private mutation in case 2 with missing incisors and canines revealed *RNF111* as a potential candidate gene. A missense mutation within *RNF111* (*ring finger protein 111*, c.1895A>C, p.Gln632Pro) with a predicted deleterious effect was found homozygous mutant in case 2, and this mutation was absent in cases 1 and 3. RNF111 was previously identified as a modulator of the transforming growth factor (TGF)-β/NODAL signaling pathway [67,68]. Through this interaction, RNF111 enhances TGF-β target gene transcription. Therefore, RNF111 plays a critical role in the induction of mesoderm and head development during embryonic development [67]. The identified missense mutation within *RNF111* may be a very likely candidate with an effect on incisor and canine development in this Fleckvieh cattle family and may contribute to the higher expressivity of the severe tooth phenotype in case 2. 

## 5. Conclusions

The missense mutation g.85716041G>A within the *EDA* gene was strongly associated with the absence of seromucous nasolabial and mucous tracheal and bronchial glands, reduced hair follicle density and partly degenerated hair follicles unable to produce hair, as well as malformed and reduced numbers of teeth. There was no evidence for further mutations in the candidate genes of the *EDA–EDAR–EDARADD* pathway. We propose that the present G227R EDA mutation in cattle has not functionally destroyed the multimerization potential of the collagen domain of recombinant EDA1 but has reduced its thermostability. Therefore, mutations affecting the first part of the collagen domain of EDA can be assumed to be more detrimental than those affecting the second part. RNF111 may be a possible candidate for the apparent differences in tooth development between the three cases and could explain the differences in the expression of the tooth phenotypes.

## Data Availability

The data supporting the study can be found in the Supplementary Materials and the raw sequencing data are openly available in the NCBI BioProject at PRJNA350739.

## Supplementary Materials

The following are available online at https://www.mdpi.com/article/10.3390/genes15010008/s1: Table S1. Phenotype and histopathological findings in previously reported cases with X-linked hypohidrotical ectodermal dysplasia (XHED). Table S2. Breed of the dam, number of teeth present and relationships among cases of previously reported cattle with X-linked hypohidrotic ectodermal dysplasia (XHED). Table S3. Number of animals used in this study. Distribution of the EDA:g.85716041G>A genotypes in 63 animals from the dairy farm with the three cases ascertained and 300 animals from different farms and breeds are given. Table S4. Primer sequences for the amplification of *EDA*, *EDA2R* and *WNT10A*. Primer pairs, amplicon size (AS) in base pairs (bp) and the annealing temperature (AT) are given. Table S5. Primer sequences used for validation of the *EDA*-associated mutation. The EDA:g.85716041G>A variant was validated using a restriction fragment length polymorphism (RFLP). Primer pairs, amplicon size (AS) in base pairs (bp), annealing temperature (AT), restriction enzyme and incubation temperature (IT) are given. Table S6. Development of body weight and daily weight gain of cases 1 and 2 in comparison with data from Fleckvieh. Comparison of the weight development of affected calves with those of male and female Fleckvieh calves according to Pichler and Frickh (2000) [62] and Becher et al. (2012) [44]. Figure S1. Gene models and protein domains for *EDA*, *EDA2R* and *WNT10A*. (A) *EDA* gene model with the exon–intron structure depicting previously reported mutations associated with hypo- or anhidrotical ectodermal dysplasia in cattle. (B) *EDA2R* gene model and (C) *WNT10A* gene model with exon–intron structures. Figure S2. Bull calf (case 1) few days after birth. At this age the calf had no hairs on its head, inner and outer sides of ears, dorsal neck, back and feet. The skin of its head also appeared rough and scaly, and the muzzle was dry. A foal blanket prevented the animal from freezing. Figure S3. Development of the tail tassels of case 1 at the age of 17.5 months (A), of case 2 at the age of 11.5 months (B) and of case 3 at the age of 7 months (C). The tail tassels only were 1/3 of the normal length at all ages up to 1.5 years of age. Figure S4. Cases 1 and 2 at the age of 7 months. All parts of the bodies of cases 1 (A-1, A-2) and 2 (B-1, B-2) were covered with normal looking, fine, soft and curly hairs except for the ears and the dorsal area from the neck to the shoulders. Figure S5. The horns (A-1) and a claw (A-2) of case 1 at the age of 17.5 months, the horns (B-1) and a claw (B-2) of case 2 at 11.5 months and the horns (C-1) and a claw (C-2) of case 3 at the age of 7 months. These structures developed normally both morphologically and histologically in the three cases. Figure S6. Case 1 at the age of 17.5 months (left) and case 2 at the age of 11.5 months (right). The coat of case 1 had lost its curliness at this age. The coat of case 2 retained curly hairs until this age but in an attenuated fashion, as in the seventh month of life. Figure S7. Sections of skin biopsies of cases 1 and 2. The neck region of case 1 (A) at the age of 10 weeks with several hairless follicles (arrows) with slight follicular hyperkeratosis. There were normal sebaceous glands and sweat glands (asterisks) with dilated lumina. The lateral neck region at the age of 10 months of case 1 (B) with a fragmented hair shaft within a dilated infundibulum. Section of skin biopsy from the head of case 2 at the age of 4 months with underdeveloped hair follicles and follicular and epidermal orthokeratotic hyperkeratosis. Hematoxylin and eosin stain (H&E stain), ×100. Figure S8. Representation of the chromosomes of case 1. The metaphase showed normal karyotypes without abnormalities. The X-chromosome is normally shaped. Figure S9. cDNA-amplicons of exon 1 to exon 9 of the *EDA* gene. The amplicons of cases 1, 2 and of the control animal were 818 bp in size and did not differ in magnitude from each other.
