# Supplementary material for: A Missense Mutation in the Collagen Triple Helix of EDA Is Associated with X-Linked Recessive Hypohidrotic Ectodermal Dysplasia in Fleckvieh Cattle
Source: Genes (Basel). 2023 Dec 20;15(1):8. doi: 10.3390/genes15010008 (PMC10815684; doi:10.3390/genes15010008)
Supplement: Supplementary file 1 [file genes-15-00008-s001.zip › Table S1.pdf]

Table S1. Phenotype and histopathological findings in previously reported cases with X-linked hypohidrotical ectodermal dysplasia (XHED) in cattle.

References	Number of animals	Muzzle	Hair coat description	Histopathological findings
Drieux et al., 1950 [48]	1		First hairless on the entire body<br>2.5-4 months old it developed sparse, bristly and nest-like hairs<br>4th month hair development on the neck, forehead, nape, back, loin, backside and later the lower areas of the body	*Epidermis* thin (2-3 cell layers)<br>*Hair* each portions are present but do not reach the skin surface<br>*Papillae* do not contain the vascular network<br>*Sweat glands* without executing channel, cystic degeneration
	2		Identical to case 1	
	3		First hairless on the entire body<br>15 days old very light fluff of hair over the entire body surface	
Rieck, 1985 [3]	1		Case identical to these of Drieux et al. (1950) [48]	
Wijeratne et al., 1988 [4]	1			
	2			
	3		First days of life severe bilaterally symmetrical hypotrichosis, thin coat of fine short silky hairs on the face, neck, ears, thorax, along spinal region, end of the tail, normal hair on abdominal region	*Epidermis* normal<br>*Hair* selective loss of large thick medullated<br>*Follicles* telogen alone<br>*Sweat glands, sebaceous glands, arrector pili muscles* normal
	4		Identical to case 3 and small tuft of hair on the forehead	Identical to case 3
Braun et al., 1988 [5]	1	Xeric	At birth hairless except for a few tufts of hair distributed<br>Some months old, fine fluffy coat on the whole body	*Epidermis* hyperkeratosis<br>*Hair* the shaft did not achieve the surface in the majority<br>*Follicles* reduced to half the normal follicle density<br>*Sweat glands* dilated
Drögemüller et al., 2001 [6]<br>Drögemüller et al., 2002 [7]	1	Dry	At birth and later in life, thinnest hair coat, no hair on the face or upper part of the neck	

	2	Dry	Few and short hair on the head, ear pinnae, neck, back and tail, very sparse hair around the eyes and on the pinnae, hairless on the backs of the ear pinnae, few and short eyelashes and vibrissae, deficiency of the hair coat was more severe in newborn and younger animals with development of fine hairs in older ones	*Follicle and appendages* absent on the backs of the ears, hair follicle density reduced on the inner side of the ear and the ventral abdomen<br>*Sweat glands* reduced on the head<br>*Eccrine nasolabial glands* absent
	3	Dry	Identical to case 2	*Epidermis* hyperkeratosis<br>*Hair* bulbs small and poorly differentiated<br>*Follicles and appendages* reduction, marked on neck<br>*Eccrine nasolabial glands* absent
	4	Dry	Identical to case 2, little more hair on the head and neck	
Drögemüller et al., 2002 [8]<br>Drögemüller et al., 2003 [9]	1	Xeric	At birth hairless<br>3 months old sparse and short hair on the back and lateral body walls	*Epidermis* thin, hyperkeratosis, hyperplasia<br>*Follicles* reduction, atrophic<br>*Sweat glands* reduced density, dilated<br>*Eccrine nasolabial, tracheal, bronchial glands* complete absence
Seeliger et al., 2005 [10]	1	Xeric	2 years old, areas of alopecia on the head, around the eyes and ears	*Epidermis* hyperkeratosis, hyperplasia<br>*Sweat glands* lacked<br>*Respiratory system* complete lack of mucous glands<br>*Nasal cavity and proximal trachea* increased number of intraepithelial and interstitial leucocytes, metaplasia of the epithelial lining with loss of cilia and goblet cells
Drögemüller et al., 2006 [11] Barlund et al., 2007 [12]	1		2 days old extremely fine hair covered the entire body, normal hair only on the inner margin of the ear pinnae, distal parts of extremities, and tip of the tail	*Epidermis* foci of secondary necrosis<br>*Follicles* few anagen, hypoplastic<br>*Sweat glands* reduced density
Gargani et al., 2011 [16]	1		Short hair coat on the head, around eyes, tail, hairless on neck, flanks, ventral abdomen	
	2		Identical to case 1	

Karlskov-Mortensen et al., 2011 [13]	1	At 7 months of age generalized symmetrical short hair coat, hairless around the eyes, margins of the ear pinnae	*Epidermis* hyperplasia, perivascular dermatitis, dilation of some lymph vessels, mononuclear and polymorphonuclear infiltrates<br>*Follicles and appendages* reduction<br>*Sweat glands* dilation<br>*Eccrine nasolabial, tracheal, bronchial glands* complete absence
	2	Stillborn identical to case 1	*Epidermis* hypoplasia of interfollicular and infundibular<br>*Hair* hypoplasia, most in telogen stage<br>*Follicle and appendages* reduction, orthokeratosis
	3	At 1 week of age identical to case 1 but hair coat was more developed on the limbs	Identical to case 2<br>*Epidermis* acute suppurative inflammation of the dermis of the left carpus<br>*Eccrine nasolabial glands* absent
	4		
Ogino et al., 2011 [14]	1	At birth hairlessness on entire body except for a part of the pinna and distal parts of the extremities	*Follicles and adnexal glands* underdevelopment
	2	Younger than two months hairlessness on entire body except for a part of the pinna	
Ogino et al., 2012 [15]	1	At birth hairlessness on most of its body, except for the distal parts of extremities	
