# Supplementary material for: A Missense Mutation in the Collagen Triple Helix of EDA Is Associated with X-Linked Recessive Hypohidrotic Ectodermal Dysplasia in Fleckvieh Cattle
Source: Genes (Basel). 2023 Dec 20;15(1):8. doi: 10.3390/genes15010008 (PMC10815684; doi:10.3390/genes15010008)
Supplement: Supplementary file 1 [file genes-15-00008-s001.zip › Table S2.pdf]

Table S2. Breed of the dam, number of teeth present and relationships among cases of previously reported cattle with X-linked hypohidrotical ectodermal dysplasia (XHED).

References	Breed of the dam	Case numbers	Incisivi	Premolars		Age at examination of the teeth	Ancestry
				Upper jaw	Lower jaw		
Drieux et al., 1950 [48]	Normande-Maine-Anjou	1	0	2	0	Six weeks, six month	Full sibling to case 2
		2	0	0	0	Five weeks	Full sibling to case 1
		3	0		0	Three days	Descendant of the maternal half-sister to case 1 and 2
Rieck, 1985 [3]	German Holstein x Holstein Frisian	1	0	4	1	Three month	
Wijeratne et al., 1988 [4]	Holstein Frisian	1	0				Full sibling to case 2
	Holstein Frisian	2	0				Full sibling to case 1
	Holstein Frisian	3	0			Six days	Descendant of the maternal half-sister to the mother of cases 1, 2 and 4
	Holstein Frisian	4	0			Four days	Maternal half-brother of case 1 and case 2
Braun et al., 1988 [5]	Fleckvieh x Red Holstein	1	1	2	5	At birth, Eleven months	
Drögemüller et al., 2001 [6] Drögemüller et al., 2002 [7]	German Holstein	1	0	4	4	Younger than three months	Maternal half-brother of cases 2, 3 and 4
		2	0	5	3	Younger than 2.5 years	Maternal half-brother of cases 1, 3 and 4
		3	0	4	4		Maternal half-brother of cases 1, 2 and 4

		4	0	4	2		Maternal half-brother of cases 1, 2 and 3
Drögemüller et al., 2002 [8] Drögemüller et al., 2003 [9]	Red German Holstein	1	0	2	0	Three months	
Seeliger et al., 2005 [10]	German Holstein	1	0			Two years	Maternal half-brother of cases 1, 2, 3 and 4 which were described by Drögemüller et al. (2001), Drögemüller et al. (2002)
Drögemüller et al., 2006 [11] Barlund et al., 2007 [12]	Red Angus-Charolais-Simmental crossbred	1	0	4	1	Two days	
Gargani et al., 2011 [16]	Holstein Frisian	1	0				Full sibling to case 2
		2	0				Full sibling to case 1
Karlskov-Mortensen et al., 2011 [13]	Danish Red Holstein	1	0	2	2	Seven months	Maternal half-brother of case 3
		2		2	1	Stillborn	Descendant of the maternal half-sister to case 1 and 3
		3	2	2	2	One week	Maternal half-brother of case 1
		4					Descendant of the grand-grandmother of cases 1 and 3
Ogino et al., 2011 [14]	Holstein	1	0	2	2	Eight days	Maternal half-brother of case 2
	Holstein	2					Maternal half-brother of case 1
Ogino et al., 2012 [15]	Japanese Black	1					
