# Supplementary material for: A Missense Mutation in the Collagen Triple Helix of EDA Is Associated with X-Linked Recessive Hypohidrotic Ectodermal Dysplasia in Fleckvieh Cattle
Source: Genes (Basel). 2023 Dec 20;15(1):8. doi: 10.3390/genes15010008 (PMC10815684; doi:10.3390/genes15010008)
Supplement: Supplementary file 1 [file genes-15-00008-s001.zip › Table S3.pdf]

Table S3. Number of animals used in this study. Distribution of the EDA:g.85716041G>A genotypes in 63 animals from the dairy farm with the three cases ascertained and 300 animals from different farms and breeds are given.

Origin		Sex			Breed	Wildtype	Heterozygous	Hemizygous affected males
		male	female	unknown		G/G<br>G/-	G/A	A/-
Dairy farm where cases were born	Affected	3			Fleckvieh			3
	Mandatory carrier (dams of cases)		3		Fleckvieh		3	
	Other animals		57		Fleckvieh	55	2	
Animals from other farms and breeds		96	97		Fleckvieh	193		
		6	3	6	Holstein	15		
			15		Limpurger	15		
		1	1	11	Limousin	13		
			12		Angus	12		

12			German Brown	12
1	11		Charolais	12
	11		Blonde d´Aquitaine	11
3	6		German Yellow	9
2	2	4	Salers	8
