# Supplementary material for: A Missense Mutation in the Collagen Triple Helix of EDA Is Associated with X-Linked Recessive Hypohidrotic Ectodermal Dysplasia in Fleckvieh Cattle
Source: Genes (Basel). 2023 Dec 20;15(1):8. doi: 10.3390/genes15010008 (PMC10815684; doi:10.3390/genes15010008)
Supplement: Supplementary file 1 [file genes-15-00008-s001.zip › Table S4.pdf]

Table S4. Primer sequences for the amplification of *EDA*, *EDA2R* and *WNT10A*. Primer pairs, amplicon size (AS) in base pairs (bp) and the annealing temperature (AT) are given.

**Primer (exon/UTR) (F=forward, R=reverse)**	**Sequence (5′ - 3′)**	**Length**	**AT (°C)**	**AS (bp)**
gEDA_Exon1_F	GAGAGGAGCTGCACATGAGG	20		
gEDA_Exon1_R	GTCCAGCCGTCGAACTTG	18	60°C	671
gEDA_Exon9_F	AATCCCTTCTTGTTGCCTCTC	21		
gEDA_Exon9_R	GATCAGTGAGAGTGGGAAGACC	22	60°C	798
X_gEDA_5UTR_F	CTGGGTCTCCTTTTCTTTTGG	21		
X_gEDA_5UTR_R	GCAGCTACTGTCCTGCCATC	20	60°C	718
X_gEDA_3UTR_F	TGGGGTAGGATGGTAATGTTG	21		
X_gEDA_3UTR_R	TGGTGACTCTTCTGTGCTGTG	21	60°C	794
gEDA2R_Exon1_F	CAGTGTCCATTAGTCCCATAGC	22		
gEDA2R_Exon1_R	CCCTCTAGGAAGCCATCTCTC	21	60°C	723
gEDA2R_Exon2_F	GCTCAGACCCAAGTTTCAAGG	21		
gEDA2R_Exon2_R	GCATCCCACAAGGTATAAAGAAC	23	60°C	665
gEDA2R_Exon3_F	TCCACAGGTTAAACAGAGCTCA	22		
gEDA2R_Exon3_R	GGGAGGTCAGTCACTATAGGC	21	60°C	877
gEDA2R_Exon4_F	TGACTGCCATACTCTCCAACC	21		
gEDA2R_Exon4_R	GGCACCTGTGAGACAAGAAAG	21	60°C	546
gEDA2R_Exon5_6_F	GAGGAAGTCCAGACCCAGTG	20		
gEDA2R_Exon5_6_R	TGATAACCATTGCCTGAGACC	21	60°C	760
gEDA2R_Exon7_8_F	TTTTCTCTGATCCACCACCAG	21		
gEDA2R_Exon7_8_R	TACCATTGTTGTCCCTCCATC	21	60°C	753
gEDA2R_3UTR_F	CAGTAGGGCTAGGGGTAGAGG	21		
gEDA2R_3UTR_R	AGTAACACTGGCCATGGAATAG	22	60°C	760

gWNT_Ex1_F	GAAGCACAGGCTCACTGC	18		
gWNT_Ex1_R	CCCCTCTAGTAGCCATCACTC	21	59°C	735
gWNT_Ex2_F	GTTGGAACTGAGGGATTATGG	21		
gWNT_Ex2_R	TTCTGGGTACTTCATCACAGG	21	58°C	780
gWNT_Ex3_F	CCCCTCTCTGAGTCTATGTCC	21		
gWNT_Ex3_R	AATGATAAGTTGGGGCTTCC	20	58°C	861
gWNT_Ex4_F	AAGACTTGCTGGCAGGTGAC	20		
gWNT_Ex4_R	AAAATCTGTCCTGGAGACCAC	21	60°C	637
