# Supplementary material for: A Missense Mutation in the Collagen Triple Helix of EDA Is Associated with X-Linked Recessive Hypohidrotic Ectodermal Dysplasia in Fleckvieh Cattle
Source: Genes (Basel). 2023 Dec 20;15(1):8. doi: 10.3390/genes15010008 (PMC10815684; doi:10.3390/genes15010008)
Supplement: Supplementary file 1 [file genes-15-00008-s001.zip › Table S5.pdf]

Table S5. Primer sequences used for validation of the *EDA*-associated mutation. The EDA:g.85716041G>A variant was validated using a restriction fragment length polymorphism (RFLP). Primer pairs, amplicon size (AS) in base pairs (bp), annealing temperature (AT), restriction enzyme and incubation temperature (IT) are given.

Gene	Polymorphism	Forward primer (5′-3′)	Reverse primer (5′-3′)	AS (bp)	AT (°C)	Restriction enzyme	IT (°C)
EDA	g.85716041G>A	CAACTGTTATGGGACCACCTG	TCCAGGAATCAAACCAGTGTC	328	66	NlaIV	37
