# Supplementary material for: A Missense Mutation in the Collagen Triple Helix of EDA Is Associated with X-Linked Recessive Hypohidrotic Ectodermal Dysplasia in Fleckvieh Cattle
Source: Genes (Basel). 2023 Dec 20;15(1):8. doi: 10.3390/genes15010008 (PMC10815684; doi:10.3390/genes15010008)
Supplement: Supplementary file 1 [file genes-15-00008-s001.zip › Table S6.pdf]

Table S6. Development of body weight and daily weight gain of cases 1 and 2 in comparison with data from Fleckvieh. Comparison of the weight development of affected calves with those of male and female Fleckvieh calves according to Pichler and Frickh (2000) [62] and Becher et al. (2012) [44].

Case 1			Case 2		Males: Pichler and Frickh (2000)		Females: Becher et al. (2012)	
Age in months	Body weight	Daily weight gain (g/day) in time interval (months)	Body weight	Daily weight gain (g/day) in time interval (months)	Body weight	Daily weight gain (g/day) in time interval (months)	Body weight	Daily weight gain (g/day) in time interval (months)
1							66	
2	60		93					
4	143.1		135		154			
4.5	147	1.160 (2-4.5)	143	667 (2-4.5)			156	867 (1-4.5)
5	162		153.9					
6	176	548 (4-6)	172	617 (4-6)	218	1.075 (4-6)		
7	201		200					
8	230	900 (6-8)			297	1.324 (6-8)		
9							300	1051 (4.5-9)
10	277	783 (8-10)			372	1.238 (8-10)		
12	342	1.083 (10-12)			437	1.065 (10-12)		
