# Supplementary figures and images for: A Missense Mutation in the Collagen Triple Helix of EDA Is Associated with X-Linked Recessive Hypohidrotic Ectodermal Dysplasia in Fleckvieh Cattle
Source: Genes (Basel). 2023 Dec 20;15(1):8. doi: 10.3390/genes15010008 (PMC10815684; doi:10.3390/genes15010008)

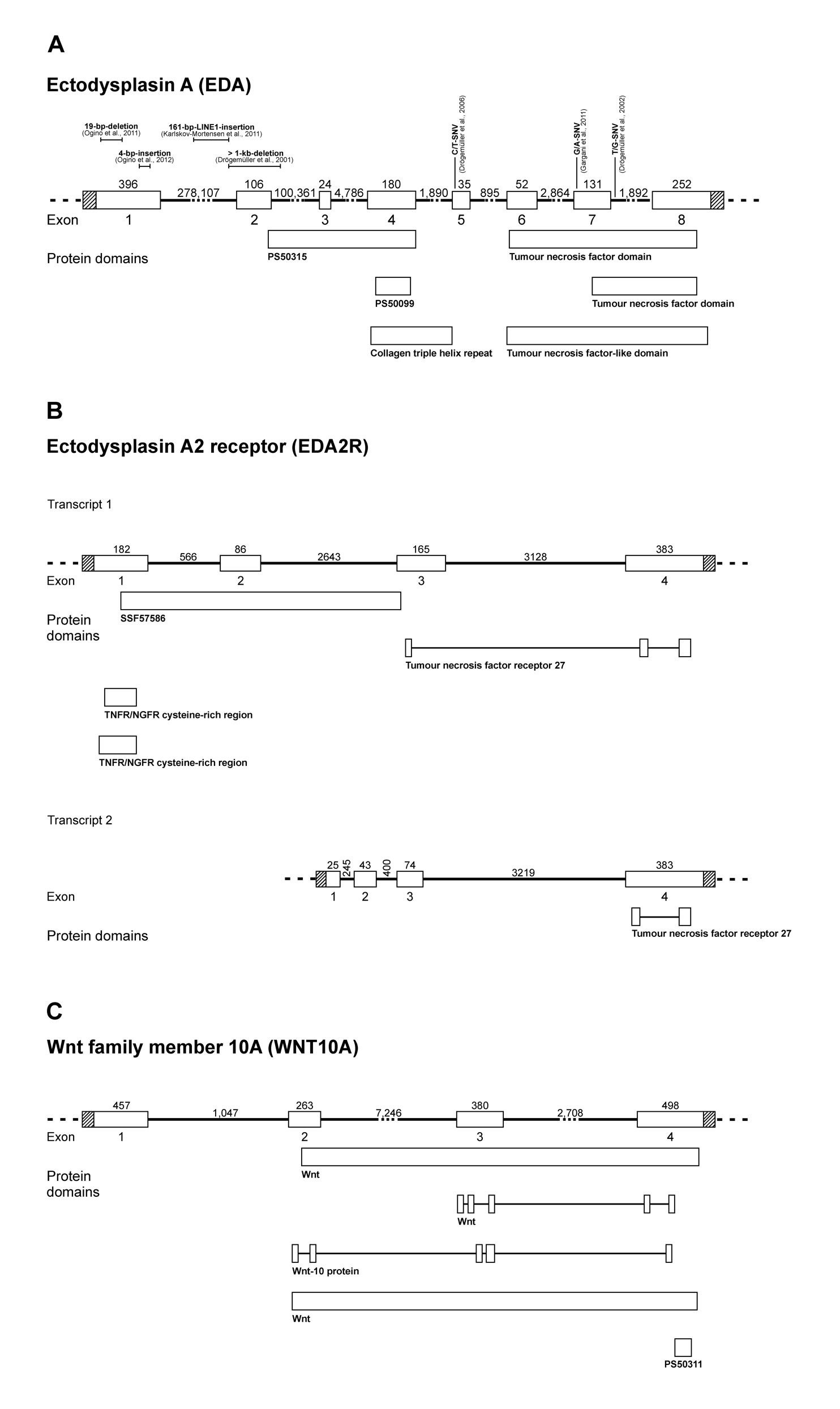

Supplement: Supplementary file 1 [file genes-15-00008-s001.zip › Figure S1.jpg]

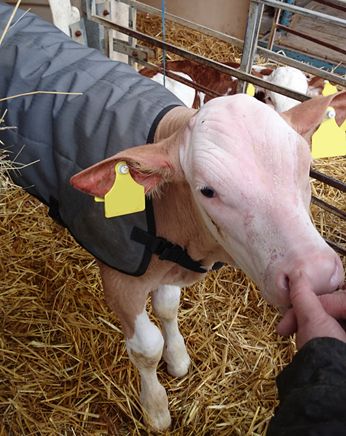

Supplement: Supplementary file 1 [file genes-15-00008-s001.zip › Figure S2.jpg]

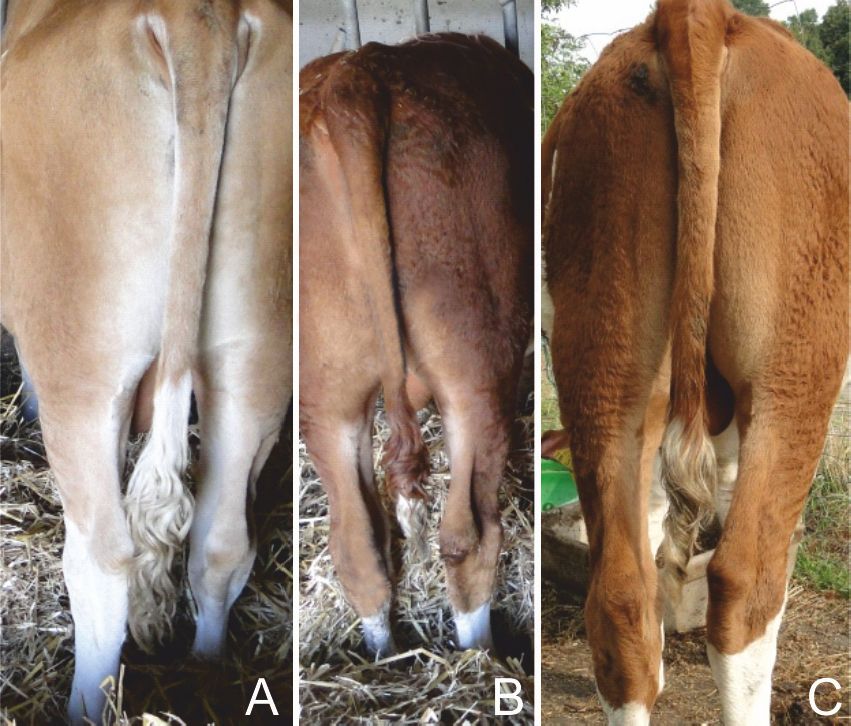

Supplement: Supplementary file 1 [file genes-15-00008-s001.zip › Figure S3.jpg]

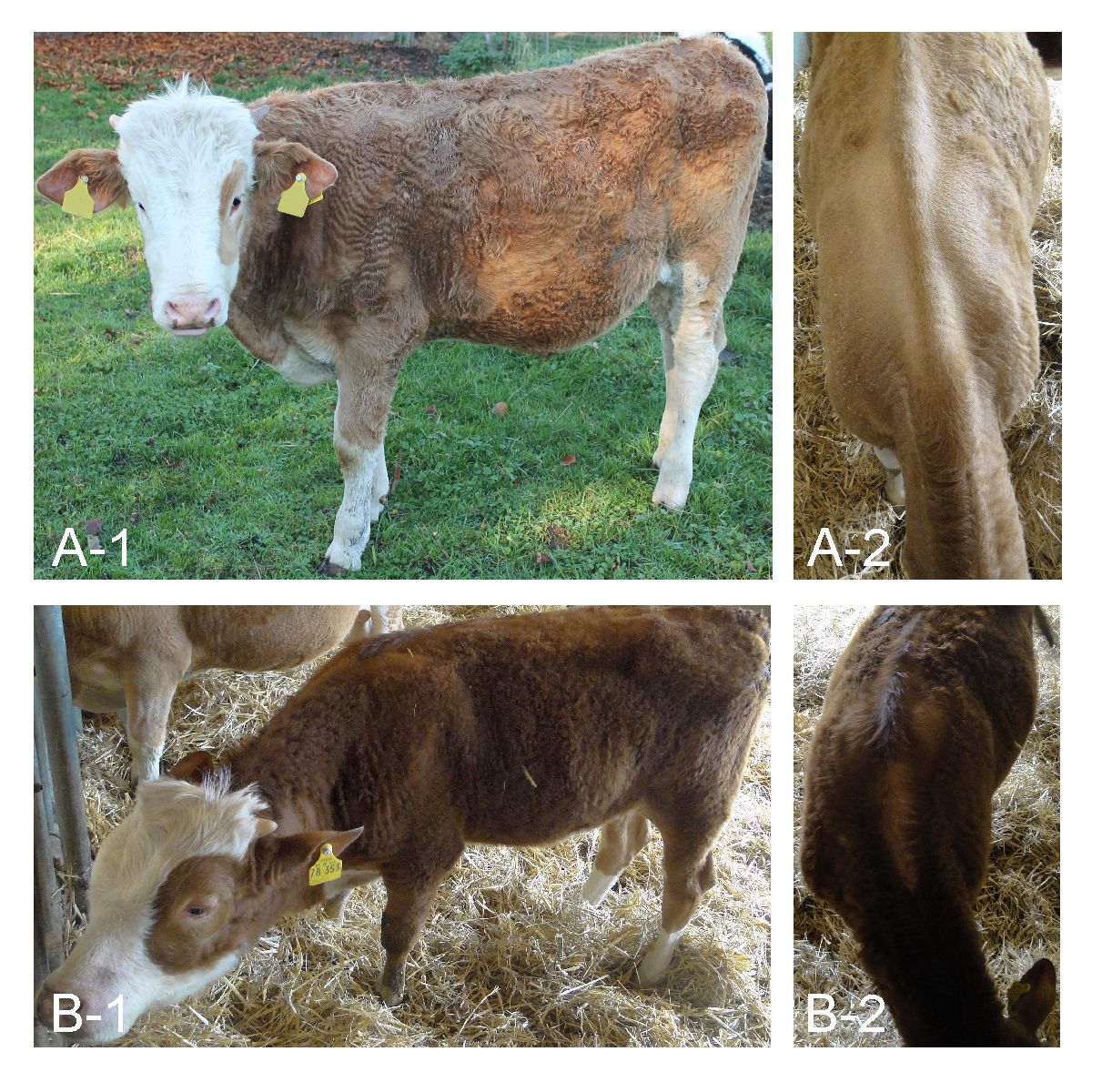

Supplement: Supplementary file 1 [file genes-15-00008-s001.zip › Figure S4.jpg]

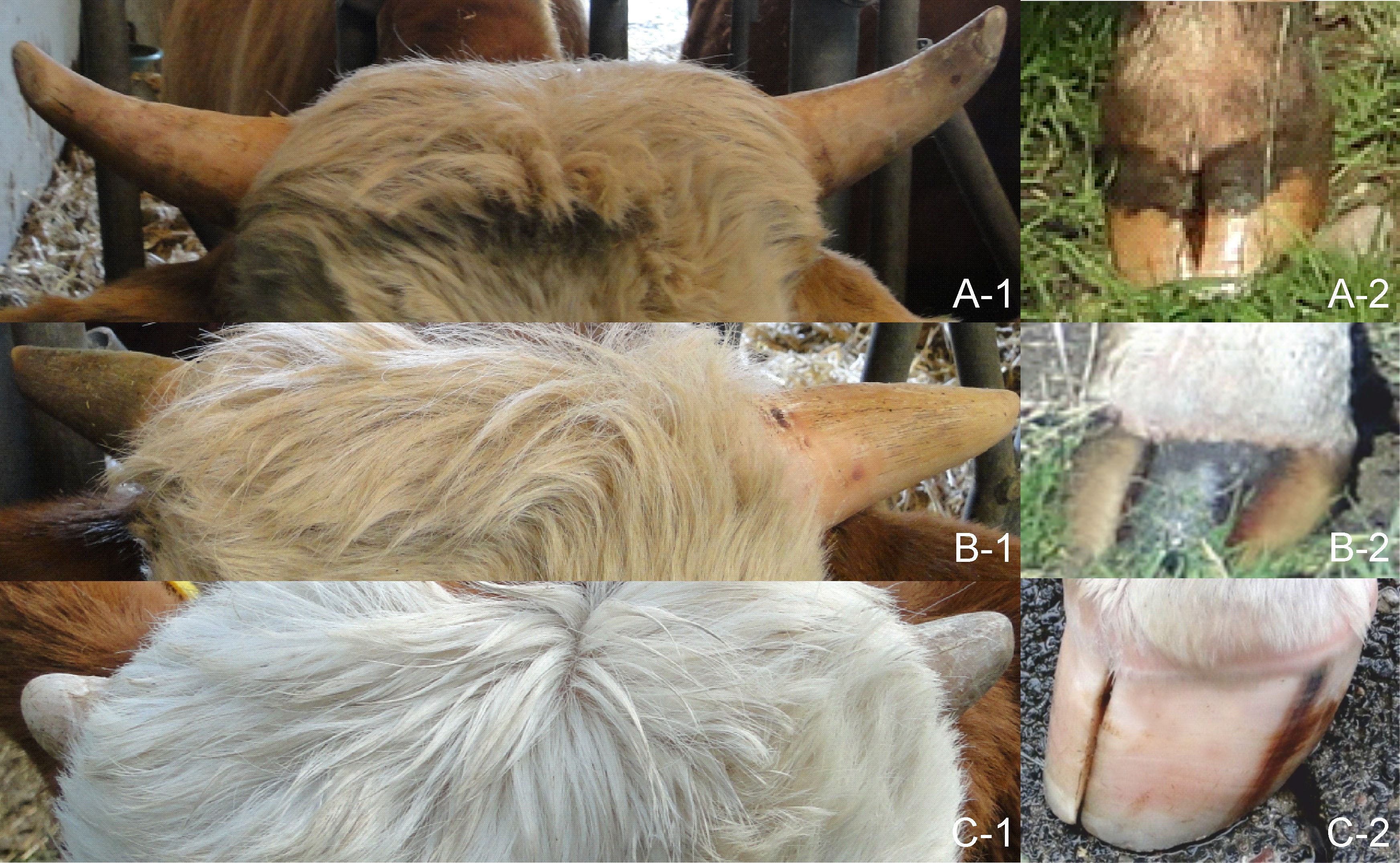

Supplement: Supplementary file 1 [file genes-15-00008-s001.zip › Figure S5.jpg]

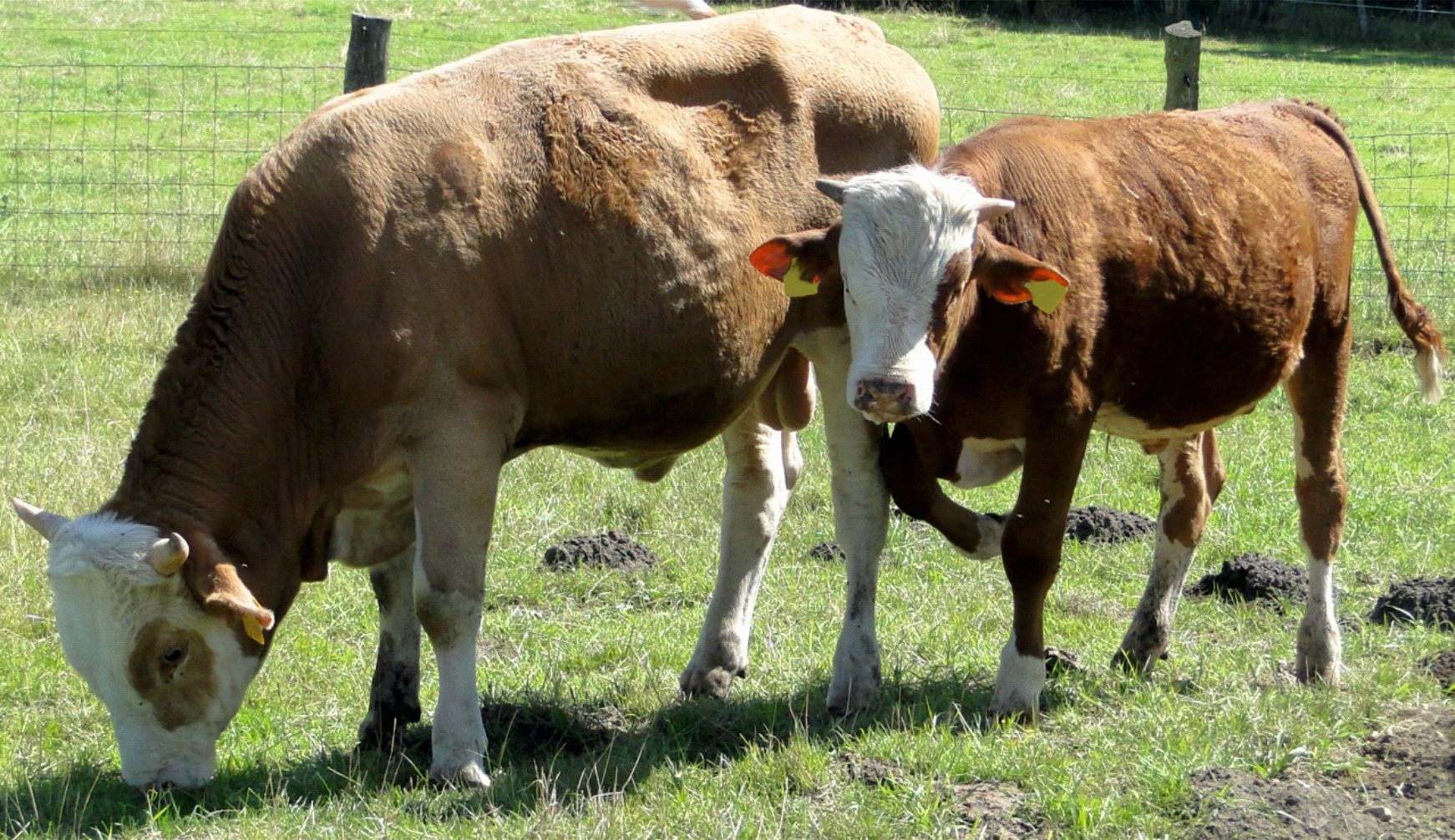

Supplement: Supplementary file 1 [file genes-15-00008-s001.zip › Figure S6.jpg]

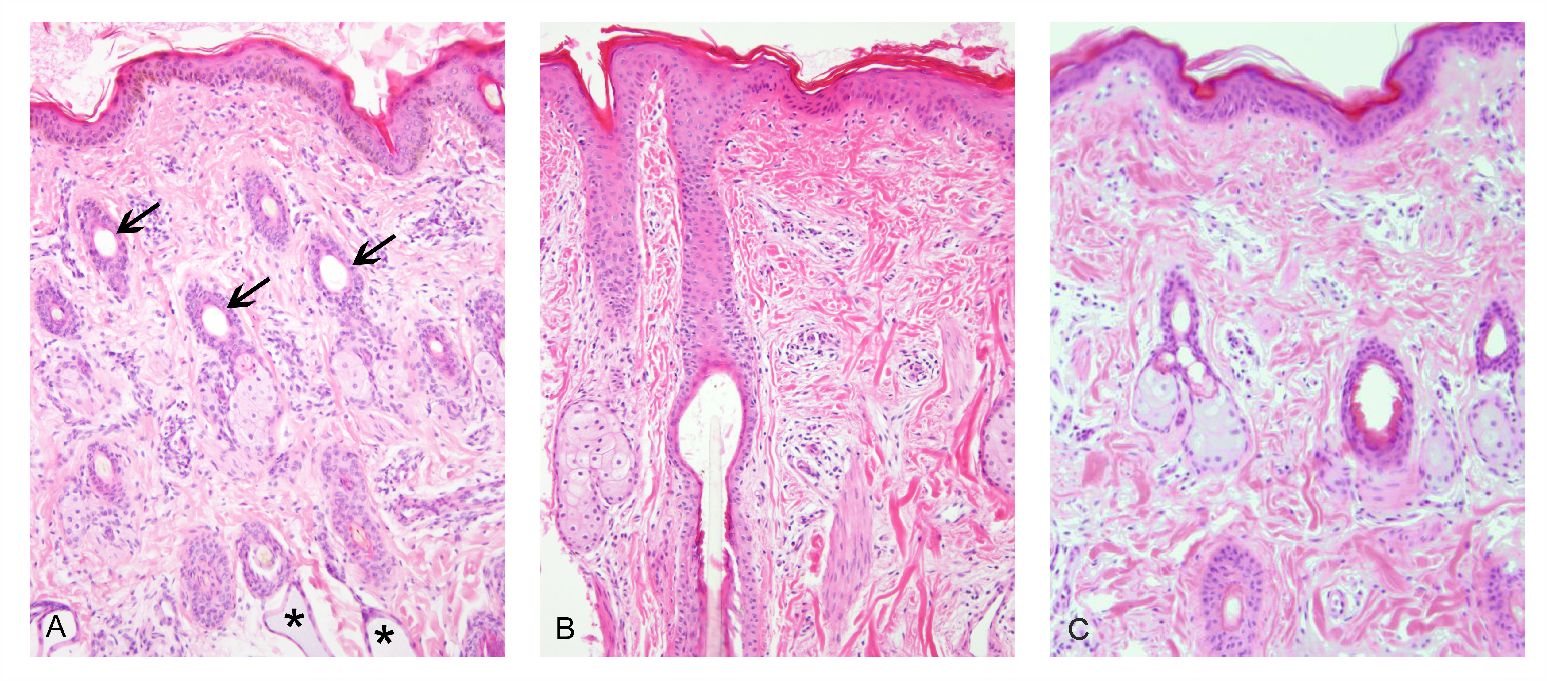

Supplement: Supplementary file 1 [file genes-15-00008-s001.zip › Figure S7.jpg]

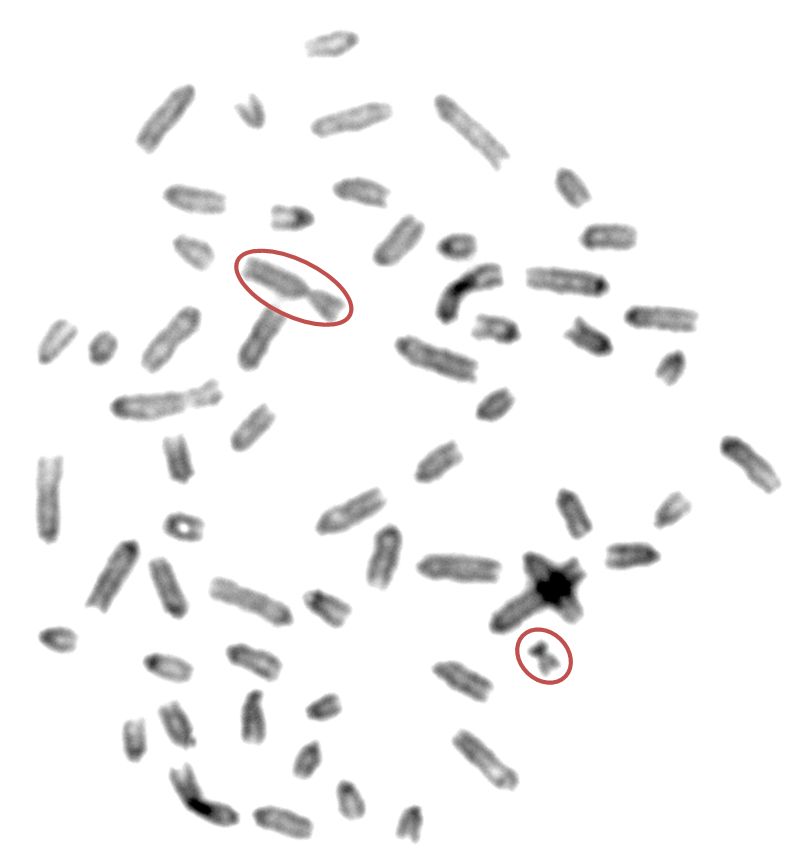

Supplement: Supplementary file 1 [file genes-15-00008-s001.zip › Figure S8.jpg]

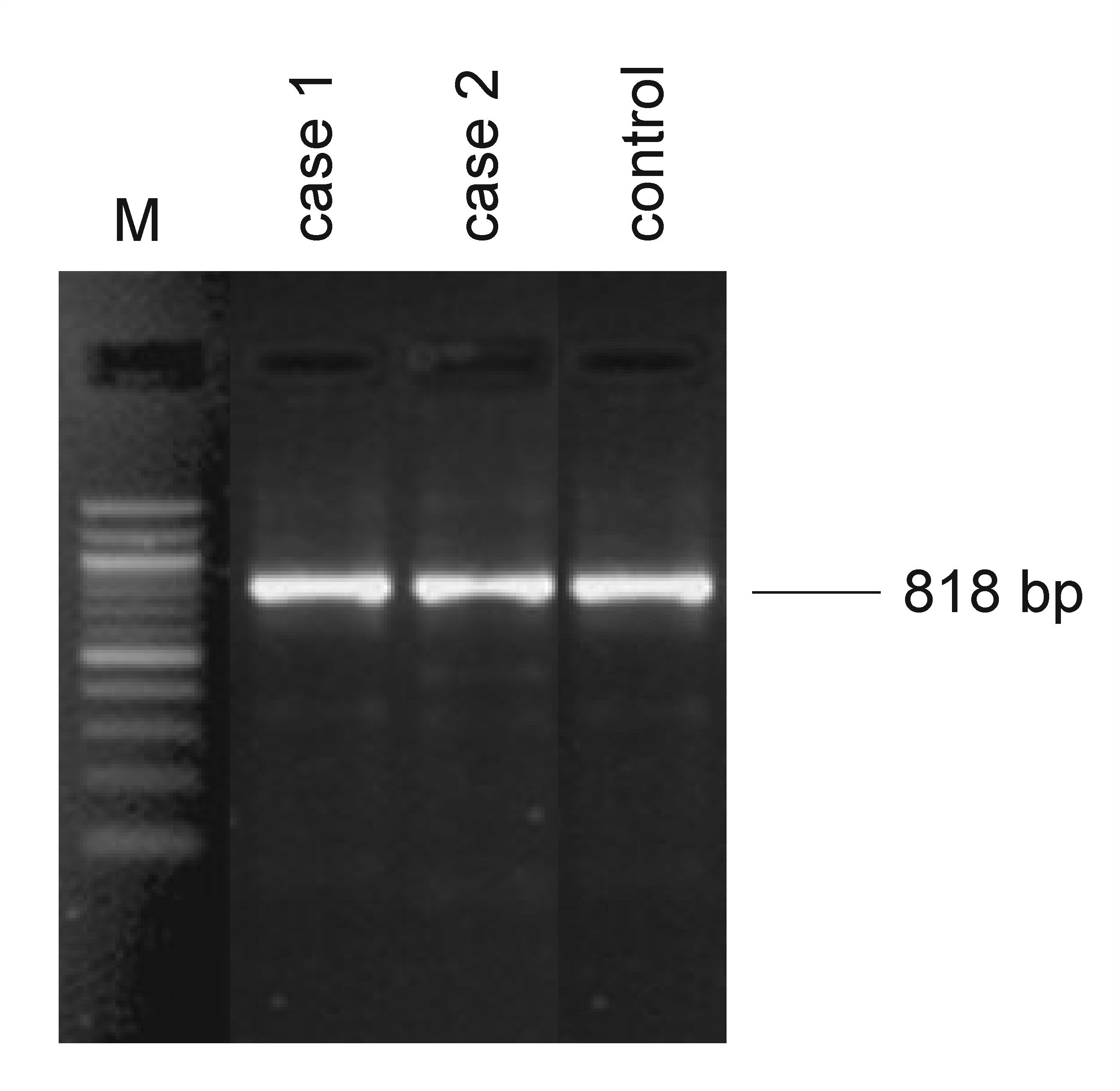

Supplement: Supplementary file 1 [file genes-15-00008-s001.zip › Figure S9.jpg]
